# Collecting and communicating perishable data in a post-disaster context: rapid research and rapid dissemination

**DOI:** 10.3389/fsoc.2023.959765

**Published:** 2023-05-12

**Authors:** Laura Gorbea Díaz, Alison Chopel, Antonio Fernós Sagebién, Lorena Bonilla Marrero, Gerardo Rivera Figueroa, Nicole Pecci Zegrí, Anohiska Cardona, Juneilis Mulero Oliveras, Luis La Santa, Paola Sánchez Rey

**Affiliations:** ^1^Puerto Rico Public and Applied Social Sciences Workshop, San Juan, PR, United States; ^2^Independent Researcher, San Juan, PR, United States; ^3^Graduate School of Business, Interamerican University of Puerto Rico, San Juan, PR, United States

**Keywords:** disaster research methods, economic equalization, disaster aid and relief, health equities, rapid research methods

## Abstract

**Context:**

Puerto Rico experienced four natural disasters in 4 years (2017–2021): Hurricanes Irma and Maria, thousands of earthquakes reaching 6.4 magnitude, and the COVID-19 pandemic. In this context, our team sought to understand the impact of disaster aid distribution on poverty and economic inequality, and their relationship to the spread of COVID-19 across Puerto Rico. Rapid research was required to ensure we could collect perishable data within this ever-changing context.

**Challenges:**

Our mixed methods design relied on both secondary and primary data. Because analyses of the former were to inform where and how to collect the latter, timing was of the essence. The data sources identified were not readily available to the public, and thus required gaining access through direct requests to government agencies. The requests coincided with a transition between administrations after an election. This resulted in unexpected delays. Once in the field, the team had to balance the rapid nature of the research with the mindful work to avoid compounding traumas experienced by participants, heightened risk for re-traumatization and fatigue, the risk of COVID-19, the digital divide, and intermittent electrical and telecommunication services.

**Adaptations:**

In response to the delayed access to secondary data, we adjusted our research question. We continued to collect data as they became available, incorporating some immediately into analyses, and cleaning and storing others for future research opportunities. To overcome ongoing trauma challenges and prevent fatigue, we recruited and hired a large temporary team, including members of communities where we collected data. By recruiting participants and co-researchers at the same time and place, we both collapsed time between these activities and increased our team's contextual competency. To adapt to challenges presented by the pandemic, we created hybrid data collection procedures where some data were collected online, and some in person, while maintaining COVID-19 protections. We used similar adaptations for dissemination.

**Lessons:**

Rapid research needs to be agile. Working within a convergence framework to investigate wicked problems had the unexpected added benefit of providing our team with a variety of disciplinary approaches which proved helpful in adapting to the changing conditions in the field. In addition to the resourcefulness of a transdisciplinary team, it is important to be willing to pivot in response to changes and to collect data where and when you can. To increase participation, opportunities need to be designed with flexibility, mindful of competing demands faced by individuals willing to collaborate. Collecting and analyzing data iteratively and utilizing local resources can enable rapid research that is rigorous and yields rich data.

**Contributions:**

Our team applied the lessons learned to structure a rapid and iterative dissemination plan. We combined member-checking with community-level dissemination, enabling us to hone findings further before presenting to policy makers and media. Rapid research creates opportunities to make data-informed program and policy adjustments when they can be most impactful. Both the media and policy makers pay closer attention to research on current events. Hence, our recommendation is to do more rapid research! The more we do, the better we will get at it, and the more accustomed community leaders, policy makers, and program designers will become to using data to inform decisions.

## 1. Introduction

In this chapter, we draw from our experience as a transdisciplinary research team designing and simultaneously implementing rapid research in a post-disaster context. Using the convergence framework for transdisciplinary research (Peek et al., 2020), three scholars from public health, applied anthropology and economics, came together as co-principal investigators (Co-PIs) to ask: How did the disbursement of disaster aid after the 2017 hurricanes impact relationships between hazard damages, poverty, economic equality, and population vulnerability to COVID-19 in Puerto Rico? In response to the challenges of the disaster context, the Co-PIs actively prioritized ethical engagement of participants and incorporated modular-like agility into the design of the methodology. Both were key to meeting research goals of providing timely insights to communities, contributing policy recommendations to government agencies, and sharing lessons learned and remaining questions with other researchers. This chapter begins with some brief, but important, context. We, then, summarize the methodology and practices set out by the Co-PIs in the original research, before we go on to reflect on the lessons, challenges, and benefits encountered in conducting and disseminating rapid disaster research aimed at introducing change across recovery systems. Original research findings have been published in depth elsewhere (Chopel et al., 2021).

### 1.1. Research context

In the Summer of 2020, with the global COVID-19 pandemic on the rise, the University of Colorado Boulder's Natural Hazards Center, with funding support from the Centers for Disease Control and Prevention and the National Science Foundation, announced a call for rapid research that would assist in improving understanding of the public health impacts and actions needed to inform responses to natural hazards across the US territories. This chapter reflects on a rapid research study funded to meet the goals of this special call.

At the time, the three principal investigators lived, and two were born and raised, in Puerto Rico, an unincorporated territory of the United States (US) located in the Caribbean Sea, that had experienced in the span of 5 years a political-economic crisis, two Category 5 hurricanes (Irma and Maria, September 2017), thousands of earthquakes ranging in magnitude and reaching up to 6.4 (December 2019 to early 2020) followed by the pandemic (COVID-19, with first reported cases in early March 2020). Prior to the hurricanes, metrics for poverty and economic inequality in Puerto Rico were higher than any jurisdiction in the US (Colón, 2021): with the proportion of children growing up in high poverty areas being six times that of the US (Backiel, 2015). The Puerto Rico government bankruptcy of 2015 had been used as justification for the gradual dismantling of Puerto Rico's healthcare system and implementation of austerity measures.

The compound disasters (Wachira, 1997) laid bare the different and unequal treatment from the US government (Willison et al., 2019). Though historically high levels of federal disaster aid were approved for Puerto Rico after the hurricanes, historically low proportions of aid had been disbursed by the time our team began its research, almost 4 years after the disasters. As of March 2021, only 27% of the over 67 billion allocated dollars had been disbursed (Willison et al., 2019; COR-3, 2021), and only 26% of FEMA funds sent to Puerto Rico had been disbursed to municipalities (Ruiz-Kuilan, 2021). Delays in the distribution of disaster aid occurred in the context of pre-existing economic, social and health inequities that can be traced to the structural violence of racio-colonial governance (Bonilla, 2020). The island became an exemplar of where colonialism is arguably the most significant sociocultural determinant of health and health inequities (Bonilla, 2020; Garriga-López, 2020; Ramos et al., 2022).

In light of scholarship that indicated that current mechanisms for federal disaster aid and recovery correlated with accelerated economic inequality and increased poverty in the US (Howell and Elliott, 2019), the proponents of the research wondered if the same trends would be true for Puerto Rico. Early evidence of the health costs of cascading disasters in Puerto Rico found that “people living in poor municipalities were 60% more at risk of dying months later due to the hurricane” (Benach et al., 2019). It was hard to fathom that the much-anticipated disaster aid, once it finally started flowing, would have an additional detrimental effect on marginalized populations facing added vulnerability to the COVID-19 pandemic. The increase and acceleration of natural hazards attributable to climate change, also, made the question urgent and its implications applicable to public health policy and programming. The daily changes experienced in the post-disaster context meant that data were perishable, in particular qualitative data on perceptions and experiences of a population experiencing unusual levels of stress and trauma, which impact memory and recall. Intent on providing timely answers and recommendations to both policy makers and communities, we set out to conduct rapid research for rapid dissemination.

### 1.2. Literature review

Natural hazards and disasters, such as those described above, often reveal gaps in knowledge. The urgency and complexity of attempting to discern if and how the distribution of disaster aid might be impacting the spread of COVID-19 placed our study at the intersection of disaster research and rapid methodologies. In this section, we review the literature that informed the original study's methodology, and with the benefit of hindsight, identify overlooked aspects of rapid data gathering and dissemination.

The need to better mitigate, prepare for and respond to disasters resulting from natural hazards, including viruses, often compels scholars to reach beyond their disciplinary boundaries (Tierney, 2019; Wartman et al., 2020). The study of post-disaster transdisciplinary collaboration informed the development of a problem-focused and solutions-based framework known as convergence research (Peek et al., 2020). Convergence research can take many forms and face formidable challenges, especially when designing a common methodology that is informed by different disciplines (Lach, 2014; Peek and Guikema, 2021). In our case, the researchers brought together expertise from economics, public health and anthropology to collaboratively design the study and collect and analyze the data, and communicate findings to varied audiences. The resulting design combined quantitative analyses of existing data sets, such as health department data and social vulnerability index data that uses census data, and field research to assist in revealing underlying mechanisms.

Reviews of disaster studies point to a long history of qualitative research that has informed current understandings of the social impacts of extreme hazards on human behavior (Faas and Barrios, 2015; Donner and Diaz, 2018). Much of the earlier research was primarily event-based and exploratory. As of the 1990s, quantitative approaches to disaster studies began to enrich the conversation, incorporating a variety of data sources, some of which may not be immediately available in the emergency or post-disaster period. In more recent years, empirical approaches to disaster research increasingly use panel data, modeling, and quantitative analyses to estimate direct and indirect economic impacts of natural hazards and related disasters (Botzen et al., 2020). In our review of the literature, we found that mixed methods were used primarily in qualitative studies to analyze primary data. However, there is a need for mixed method designs that bridge the gaps between quantitative and qualitative disaster studies. Few, if any, complex problems can be understood with quantitative or qualitative findings alone, and even fewer solutions can be meaningfully informed with only one or the other.

Our research was designed to build on quantitative analyses of economic and population data sets from 1993 to 2013 that explored the relationships and behavior of poverty and economic inequality a year after an extreme natural hazard event. Smiley et al. (2018) examined the numbers of private organizations, both non-profit and for-profit, and noted that growth in the number of non-profit organizations correlated with increased poverty, with the exception of advocacy organizations. Looking at the same timespan, Howell and Elliott (2019) found that federal disaster aid was associated with increased economic inequality across all counties of the U.S. More specifically, they showed that aid increased poverty and wealth inequalities. Though these analyses identified important relationships that impacted communities' abilities to recover from a disaster, the data analyzed did not include US territories. Research by Smiley et al. (2018) coincided in identifying the need for qualitative research to provide greater understanding of the relationships observed.

Event-based disaster studies provide a wealth of insights into changing practices of cooperation, growth of communitas (Casagrande et al., 2015), the role of and re-creation of social networks in recovery and preparedness (Jones and Faas, 2017), the underlying causes and systemic reproduction of disaster, risk perception, community organization, to name a few. Recent studies have also examined the initial protection offered by social capital that is lost or wanes in the recovery period (Islam and Walkerden, 2015; Hernández et al., 2018; Talbot et al., 2020). Though these works raise important considerations, the scope and variables studied in each case make it difficult to specifically address the phenomena observed by large scale quantitative analysis such as those of Smiley et al. (2018). By looking at the economic and population data from before and after the cascading natural hazards in the context of Puerto Rico, our research sought to add a new geographical and social context to the findings of Smiley et al. (2018), and through exploratory research examine the relationship between event damages, aid distribution, poverty, economic inequality, and COVID-19 reported cases.

When disaster research is designed to provide rapid response to guide policy, as was the case at hand, there is an inherent tension between providing timely feedback and working to overcome limitations in sampling, methods and with time for reflexivity in the analysis (Vindrola-Padros and Vindrola-Padros, 2018; Sangaramoorthy and Kroeger, 2020). To fit the time limits, rapid research approaches have favored qualitative data collection methods (Beebe, 2001, 2014; Sangaramoorthy and Kroeger, 2020). To overcome criticism of rapid research as being “quick and dirty,” rapid research studies have incorporated the use of triangulation, local research assistants, and participatory methods (Vindrola-Padros and Vindrola-Padros, 2018). There is still limited insight, however, on how knowledge can be collaboratively and inclusively produced in a post-disaster context or during a period of crisis and still fit into a rapid timeline. Other challenges cited across reviews of rapid and disaster research include earning stakeholders' trust, achieving collaboration across a variety of stakeholders and limited time to train field team members (Donner and Diaz, 2018; Vindrola-Padros and Vindrola-Padros, 2018).

In recent years, rapid and disaster research have faced a variety of critiques. Noting the positionality of external disaster researchers entering the field of study to gather data, a critical review has recognized a culture gap in hazards science (Wu et al., 2022). The Natural Hazards Center inaugurated a new cultural competence online course (Wu et al., 2022) to help address this. In addition to this gap, the disaster researcher often has the difficult task of studying sensitive topics in moments when they may be generating additional burdens on populations still struggling to recover. In response to the historical and recent instances of exploitative and harmful research that have been conducted in the Caribbean, and the particular vulnerabilities that exist in a post-disaster space, there are jurisdictions in the Caribbean exploring limiting disaster research (Louis-Charles et al., 2020). In recognition of these political, social, and economic costs of disaster research, many scientists have called for more respectful and reciprocal engagement with local participants and local scientists (Gaillard et al., 2019).

Knowledge sharing in ways that are responsive and inclusive is an underdeveloped area in the literature on disaster and rapid research. Our review identified repeated references to challenges in dissemination or the need for greater attention to details in how findings are communicated across stakeholders (Vindrola-Padros et al., 2021). A review of rapid ethnographies in healthcare found only a few peer-reviewed articles addressed dissemination efforts and recommended that future researchers who do so design dissemination strategies that do not reduce the richness of the data (Vindrola-Padros and Vindrola-Padros, 2018). Within healthcare we found non-ethnographic examples of dissemination of actionable protocols and briefs that were informed by the findings (Higham et al., 2022; Walton et al., 2023). The rapid conversion of findings to action through the dissemination of protocols underscores the institutional endorsement of the research. By contrast, among researchers in disaster studies, we find repeated references to the challenge of getting research to inform changes in policy or having social scientists have a seat at the table (Oliver-Smith, 2016; Faas et al., 2020). The shared interest by both, rapid and disaster research fields in diversifying knowledge sharing to mitigate disaster impacts has led to promising advances that explore how to communicate protocols, rich data and findings using participatory engagement of communities. These efforts have noted the persistent challenge of bridging interdisciplinary discourse common in disaster studies (Agyepong and Liang, 2023). Recent research using the convergence framework in disaster risk communication in Puerto Rico shows a path forward through the disciplinary gaps using an iterative process of engagement (Davis and Gand́ıa, 2021).

Our review of both disaster and rapid research underscored the importance of identifying the positionality of the researchers in relation to the target audiences for dissemination. Rapid research on pandemic responses within the healthcare industry showed that administrative commitment was correlated with results in dissemination of findings to decision-makers and integration into policies, protocols, and processes. The challenge most commonly cited by researchers working within institutions was disseminating research beyond the institution or industry in peer-reviewed publications (Vindrola-Padros and Vindrola-Padros, 2018; Sangaramoorthy and Kroeger, 2020). The complementary challenge remains: how can disaster researchers positioned outside of an institution advance the dissemination and use of findings within relevant institutions? In the case at hand, our research was performed thanks to funding from federal agencies, heeding a call to provide rapid feedback to inform change. This chapter describes our process to advance the use of research to inform policy in the results and challenges sections.

### 1.3. Researcher positionality, reciprocity, and other ethical considerations

Natural hazards and disasters, such as those described above, often reveal gaps in knowledge. Our core team was mindful of the history of abusive research (Briggs, 2003; Ramos et al., 2022; Shamoo, 2022) that has impacted Puerto Rico and thus committed, not only to ensure the ethical treatment of participants, but also to conscientiously seek reciprocity with them and engage them in the definition of potential uses and recommendations that would emerge from the research findings. Our commitment motivated and informed the question guiding the research and placed the project within the body of critical and engaged scholarship (Low and Merry, 2010). In this section, we review the decisions made in the design and implementation that were informed by our commitment to reciprocity, ethical engagement of study participants and advocacy for policy change.

The study that we report on here was designed from within the post-disaster context. The question selected addressed the immediate concern of potential participants and collaborators, at the same time, it informed people about, and built upon, research that had been undertaken across the US. The research team's diversity extended beyond ethnic origin and lived histories to disciplines of research and practice.

Researcher positionality was communicated in invitations to collaborate in the study and in informed consent process. The research was presented to participants as a concern shared by three local social scientists for the impact the distribution of Hurricane Maria related federal assistance had on the health and preparedness of people living in Puerto Rico. The research objective was to generate knowledge to inform policy change and identify recovery strategies that worked without increasing inequalities. This locally engaged research was further described as being sponsored and funded by scientific organizations (Natural Hazards Center and National Science Foundation) and a federal agency (Centers for Disease Control and Prevention).

All primary data collection procedures were reviewed and approved by the Ethical & Independent IRB (case reference 20221–01), an independent Institutional Review Board (IRB) with a long history of reviewing public health research, an understanding of participatory research approaches, and an ability to review study designs and findings in Spanish and English. While our original intention was to use the IRB of our academic co-researcher located at a university, the pandemic added greater delays to their process timeline and threatened to delay our ability to collect primary data in line with our rapid research timeframe. Therefore, we decided to use an independent for-profit IRB that one of our research team members had worked with before. Ethical treatment of participants meant not only communicating informed consent in understandable language, but also discussing the additional protocol observed for reducing risk of COVID-19 transmission during research activities. The data gathered were anonymized prior to analysis and eventually preserved for subsequent analysis in the custody of the Puerto Rico Public and Applied Social Sciences Workshop (PR PASS Workshop), a nonprofit organization that provides technical assistance to researchers. The field team received training prior to heading to the field and had their interactions recorded and reviewed to ensure quality and corrections were made in a timely fashion.

In the research implementation stage, thirteen of the fifteen members of our extended research team were born and raised in Puerto Rico, were bilingual in Spanish and English and had lived through the cascading disasters. Of the two who did not identify as Puerto Rican, one lived there and was bilingual.

Our field research team^1^ members had to be residents or have personal connections to the towns we were investigating. Our aim in doing so was three-fold: (1) to facilitate social trust, (2) to engender reciprocity, and (3) to leave a social and economic impact in the towns where we were collecting data and discussing findings. At a time when many participants faced exhaustion from cascading and compound crises, the research team chose to humanize the concern by using local residents to assist in collecting data. The field team was trained to first show concern and solidarity for every participant prior to introducing the why and how of the research during the process of gaining informed consent. Researchers personally knew some participants, and others were referred to us by organizations, fieldwork assistants, or participants. Being and sounding local also meant the research team shared or had witnessed some of the experiences described by participants. The process, set out to frame the interview as a “conversation among neighbors and peers” was also designed to inform policy. In addition to the process of listening and bearing witness to participant stories, the research also manifested reciprocity with participants through the award of collaboration stipends and the hosting of town hall meetings to discuss preliminary findings in local community centers or restaurants.

In these town hall meetings, our stakeholders were able to see and hear first-hand how their privacy had been protected. Stories were shared using fictitious names. The town names did not appear in our disclosure materials. Descriptions of the towns were rendered in ranges to assist in anonymizing the town and its residents. In an exercise of reciprocity and in service of accountability, communication products used in these events were shared, and still are available online for participants to review and comment at www.prpassworkshop.org. Finally, all participants present at the town hall meetings were also invited to the policy seminar that was held virtually a month later. The final research report was also made available online in a Spanish translation.

Authorship of the final report was offered to research collaborators. The process for inclusion was discussed during the on boarding of fieldwork assistants. The rule of thumb discussed, recognized that a contribution substantial enough to warrant authorship could be attained through consistent participation throughout the data entry, data gathering, analysis, town hall meetings, and policy seminar. In practice, this could be achieved by a student or community collaborator active in data entry, who also participated in the town hall meetings or the policy seminar, or by a fieldwork interviewer or participant that came to the town hall meeting, expressed interest in ownership of the recommendations and came to the policy seminar. To make this offer more attractive, research assistants were offered stipends throughout the project proportionate to the tasks selected. As an additional measure for inclusivity in the generation of knowledge, research contributorship was extended to all research collaborators. Other fields are increasingly using similar approaches used in other scientific fields which increasingly cite the CRediT (Contributor RolesTaxonomy) and decide for each project how many roles and contributions are needed to attain authorship (Allen et al., 2014; Cooke et al., 2021; Myers et al., 2021). The fact that none of the research collaborators appear as co-authors has been the subject of much reflection among the co-principal investigators. Students that had been quick to join the ranks to represent populations they knew, excused themselves from joining additional data analysis or dissemination activities. The common theme among the candidates was lack of time due to the beginning of new internships or new jobs.

## 2. Methods

The guiding research question for the study was: How did the disbursement of federal disaster aid after the 2017 hurricanes in Puerto Rico impact the relationships between hazard damages, poverty, and population vulnerability to the public health risk of COVID-19 across all 78 municipalities? Mixed methods were integrated to examine dynamic relationships between hazard damages, emergency responses, recovery efforts, economic inequality, and public health vulnerability in Puerto Rico. We first investigated the relationship between poverty rates, hazard damages and disaster aid. We then conducted case studies in two municipalities, selected with guidance from our quantitative findings. The study had four specific aims which we list below:

Aim 1: Examine the changing rate of municipal poverty from 2015 to 2019 and whether damages from hurricanes Irma and Maria (2017) accelerated increases in poverty.
Aim 2: Ascertain the influence of federal disaster aid on the change in poverty rates.
Aim 3: Elucidate the relationships between hurricane damages, disaster aid, economic inequality and each municipality's ability to prepare for a public health threat, by investigating distribution of COVID-19 cases across municipalities.
Aim 4: Identify potential underlying mechanisms of dynamic relationships identified in Aims 1–3 by exploring the impacts of federal disaster aid in two municipalities, using case study methodology.

The aims were designed to have modular agility. Work pertaining to the first three quantitative aims were able to progress on their own with existing data while protocols and instruments were being developed and the independent ethics review board approval was attained. Correlation and regression models enabled analyses of municipal measures of damages, aid, poverty, economic equality, and COVID-19 burden. Results from Aim 2 guided our case study site selection for the qualitative study (Aim 4). Case studies were conducted to explore mechanisms of relationships identified at the macro level.

To integrate findings from secondary and primary data we designed an iterative approach. While seeking protocol approvals and ethical reviews, we optimized time usage by focusing on secondary data gathering and quantitative analyses. Analyses of these data would inform where and how primary qualitative data would be collected. As researchers that were practitioners in disaster recovery zone after an election that generated change in federal and state government, our data plan included several proxies to account for delays or limited access to our preferred sources of data. In the case that data sources identified were not readily available to the public, we attempted to gain access through direct requests to government agencies.

### 2.1. Study sites

The research location for all aims was Puerto Rico. For the quantitative study aims, the municipality was the unit of analysis. We compared data for all 78 municipalities in Puerto Rico. The results from Aim 2 guided our case study site selection for the qualitative study (Aim 4). We identified the range of the resulting correlations between disbursed aid and changes in poverty in all municipalities and then selected one of the three municipalities with the average correlation and the municipality with the farthest outlier correlation (which happened to be the smallest). All correlations were positive, and clustered around the averages, leading us to believe that conducting a case study in one of the three municipalities with the average correlation could potentially illuminate possible broader underlying mechanisms contributing to the observed positive relationship between federal aid distribution and increasing poverty. In the vein of appreciative inquiry, we felt a comparison between the municipality that exemplified the correlation and the municipality where federal aid seemed to have the smallest impact on increasing poverty would help identify potential mediating factors reducing the intensity of the relationships.

Since there were three municipalities with the average correlation, we were able to select two municipalities in the same peri-urban region. Primary data collection for Aim 4 was conducted in person in the two selected municipalities. In order to extend privacy and honor confidentiality agreements we referred to the sites with the fictitious names Nube and Suelo. Nube was a municipality with a population of under 40,000 people and was described by residents as “campo” (rural). Nube represented the average positive relationship between aid and poverty in PR. In Nube, over a 7 year period, the percentage of population living below poverty level (PPBPL) grew by 4%. Suelo, on the other hand, was the municipality with the smallest identified relationship between aid and change in poverty (although still a positive relationship). Its population was ~70,000 and it had both rural communities and more suburban developments. In Suelo, the PPBPL decreased by 19% over the same period.

### 2.2. Data, methodology, and procedures

The study methodology is described more in-depth elsewhere (Chopel et al., 2021). Below we summarize our sample and secondary data and data analysis procedures.

**Aim 1: Examine the changing rate of municipal poverty from 2015 to 2019 and whether damages from hurricanes Irma and Maria (2017) accelerated increases in poverty**.

Poverty was measured as the proportion of the population whose income fell below the U.S. Census poverty line. The fact that these datasets are already readily accessible made attractive for our rapid mixed methodology. We estimated the changing rate of municipal poverty by calculating the relationship between year and poverty while holding constant other changing demographic measures including: U.S. Census estimates of the total population, proportion of the population with a bachelor's degree, percent of the population below age 18 and above 65 and the Puerto Rico Department of Labor's quarterly average wage. We then calculated the relationship between year, total population, and poverty for each municipality separately. By calculating the difference between the year coefficient pre-2017 and the year coefficient post-2017, we were able to approximate how much the change in poverty rate altered after the hurricanes. Using this as a dependent variable, we examined the relationship between this alteration and hurricane damage, conceptualized as both property damages and fatalities. Hurricane property damages were approximated with the Special Hazards Events and Losses Database for the US (SHELDUSTM) non-crop property damages and fatalities were calculated by the Puerto Rico Center for Investigative Reporting.

**Aim 2: Ascertain the influence of federal disaster aid on the change in poverty rates**.

For Aim 2, we built on Aim 1's models by adding data from the FEMA and Community Development Block Grant Disaster Recovery programs on their aid distribution in each municipality. Our key independent variable for this analysis was the total disbursed aid including assistance to individual households and assistance to municipalities. This data was readily available online in an easy to use format.

**Aim 3: Elucidate the relationships between hurricane damages, disaster aid, economic inequality, and each municipality's ability to prepare for a public health threat, by investigating distribution of COVID-19 cases across municipalities**.

For Aim 3 we used Puerto Rico's Department of Health municipal COVID-19 cumulative case counts from April 2020 to April 2021. We included non-duplicated positive PCR and serology tests. We calculated correlation estimates between COVID-19 case counts and total aid disbursed, number of fatalities attributed to Hurricane Maria, total damages in dollars, and the Gini coefficient for each municipality. Raw data used was available online but required processing to arrive at the format and quantities used in our analysis.

**Aim 4: Identify potential underlying mechanisms of dynamic relationships identified in Aims 1–3 by exploring the impacts of federal disaster aid in two municipalities, using case study methodology**.

We used ethnographic observation and structured interviews (*n* = 76) to collect data. Interview guides were developed to focus on factors in multiple eco-social dimensions. Areas of interest, curiosity, and confusion for further exploration were identified by the research team in the process of discussing results of Aims 1 and 2.

### 2.3. Sample size and participants

As discussed above, we selected two theoretically advantageous municipalities for the Aim 4 case studies. Within each municipality, invitations to participate in the research were distributed on social media and randomly distributed to individuals in public spaces. Eligibility criteria for study participants included being over 18 years of age and being a resident of the municipality for at least 5 years. Using PR State Department records, we conducted stratified random sampling to invite 30 organizations to participate that were equal parts for-profit businesses, social, and advocacy nonprofits. This sampling strategy was hampered by the lack of accurate information, as 36% of organizations did not report accurate contact information or could not be otherwise found, and 24% responded late or negatively to the invitation. Efforts to secure residents as research assistants improved participation. The final sample included 20 organization-affiliated participants (four business owners, four public servants, and 12 employees or social organization members), and 56 unaffiliated residents. The poverty rates of those interviewed reflected the overall poverty rates in each municipality (see Table 1).

**Table 1:** Poverty in case study site and sample (Chopel et al., 2021).

	**Percent of the population earning below poverty level**	**Participants earning under $20,000 (%)**
Nube	50–59	58
Suelo	26–39	32

We interviewed a total of four public servants, four business owners and 71 residents (where *N* = 76, because all but three business owners were also residents). Finally, we examined correlations between hurricane damages, hurricane fatalities, disbursed aid, economic inequality, and COVID-19 cases. For further information on participant demographics by municipality (see Table 2).

**Table 2:** Participant demographic information (Chopel et al., 2021).

*N* = 73*	**Nube**	**Suelo**
Females	27	16
Males	19	11
Individuals who self-identify as LGBTQ	3	1
Percent of participants who self-identified with the 2 darkest skin tones.	6.5%	4.0%
Organizational leaders	4	4
Percent of sample earning below $20,000	57%	32%
Has lived 11+ years in the community	78%	89%
Age 21–25	30%	16%
Age 36+	70%	84%
Percent of the sample that has bachelor degree or higher	30%	30%
Percent of the sample that went to private schools (k-12)	11%	20%
Number of participants who received FEMA aid	15	9
Number of participants who received municipal aid after H. María	21	6
Number of participants who received COVID-19 municipal aid	16	4
Number of participants who received COVID-19 federal aid	4	2

*Demographic measures were collected from 73 participants. Measures were not collected from the remaining three participants (two public servants, one business owner).

### 2.4. Secondary data

For Aims 1–3, data from all 78 municipalities were used. In addition to the different sources of data cited above for each aim, in order to capture a more detailed picture of the myriad of different factors that both contribute health inequities and create higher vulnerability to health and property damage in marginalized communities, we also incorporated the Social Vulnerability Index (SVI) into our analyses. The SVI was developed by the Centers for Disease Control and Prevention for this purpose, and as defined by the U.S. Census, “The Social Vulnerability Index uses U.S. Census data to determine the relative social vulnerability of every census tract.” The SVI ranks each tract on 14 social factors and groups them into four related themes. Each tract receives a separate ranking for each of the four themes, as well as an overall ranking. The SVI can help emergency response planners and public health officials identify and map the communities that will most likely need support before, during, and after a hazardous event.

When incorporating the SVI into our research, we kept in mind that vulnerability is not a static experience where all 14 social factors remain equally relevant across time and place. Our review of the CDC's SVI index let us to use a modified version of the SVI that had been adapted to the Puerto Rican context. For example, the CDC version included the percent of non-English speaking population did not provide the same kind of information it did in the US because Spanish is the official and commonly used language across all of Puerto Rico's municipalities. Non-Hispanic, white population percentage was also not very meaningful due to the fact that nearly all residents of Puerto Rico identify as Hispanic or Latino. Thanks to the generosity of colleagues at the Vulnerable Coastal Communities Initiative (VCCI) of the Center for Community Progress, we were able to use an SVI, measure modified specifically for Puerto Rico, henceforth referred to as VCCI-SVI.

### 2.5. Data analysis

**Figure 1:**
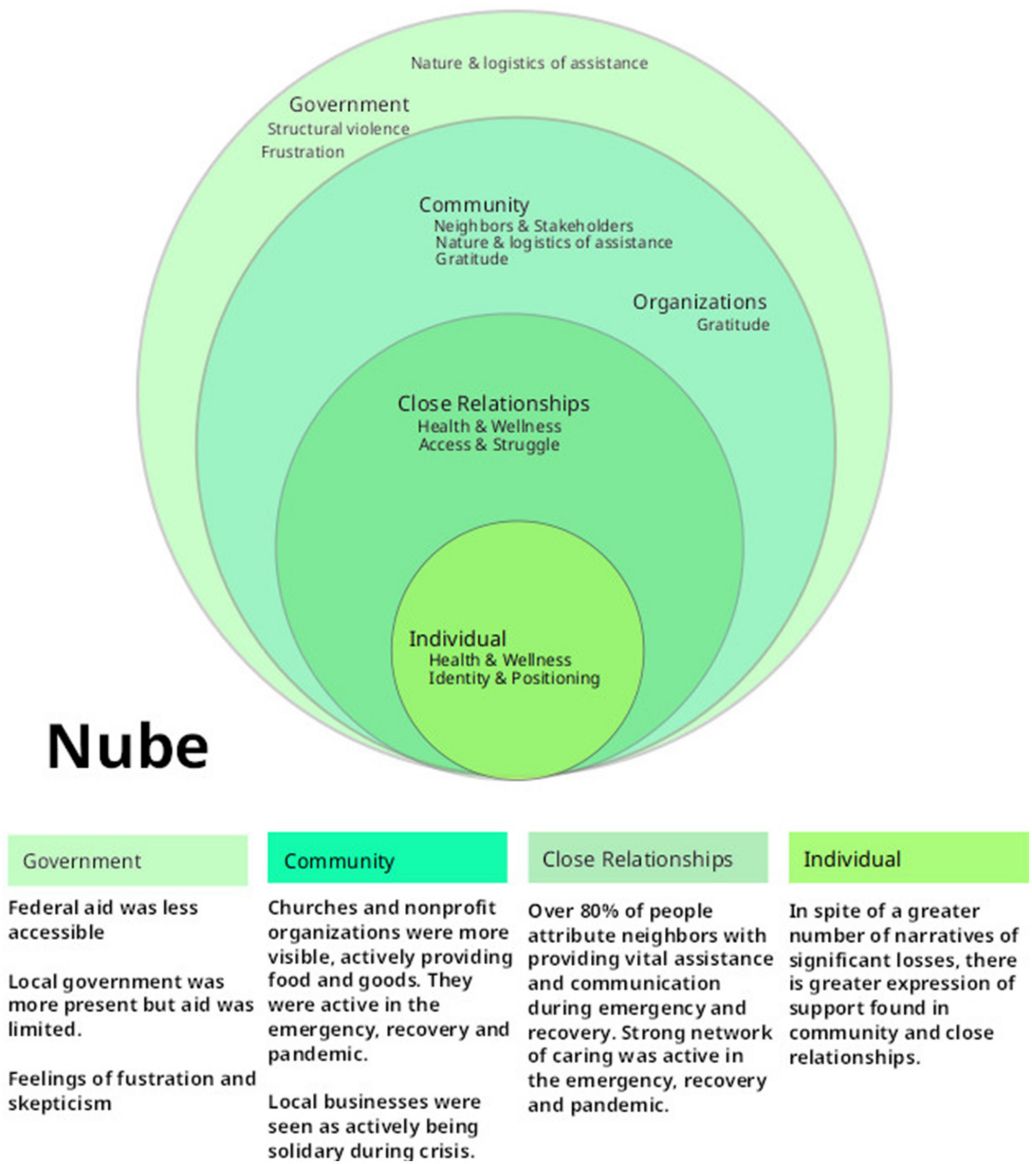
Ecosocial model of nube. This is a visual representation of actors, activities and emotions associated with disaster recovery across four dimensions of interaction. The overlapping rings communicate the interplay between the dimensions. The maps are informed by an analysis of participant evaluation of actors and services in each dimension and participant narrative analysis (Chopel et al., 2021).

**Figure 2:**
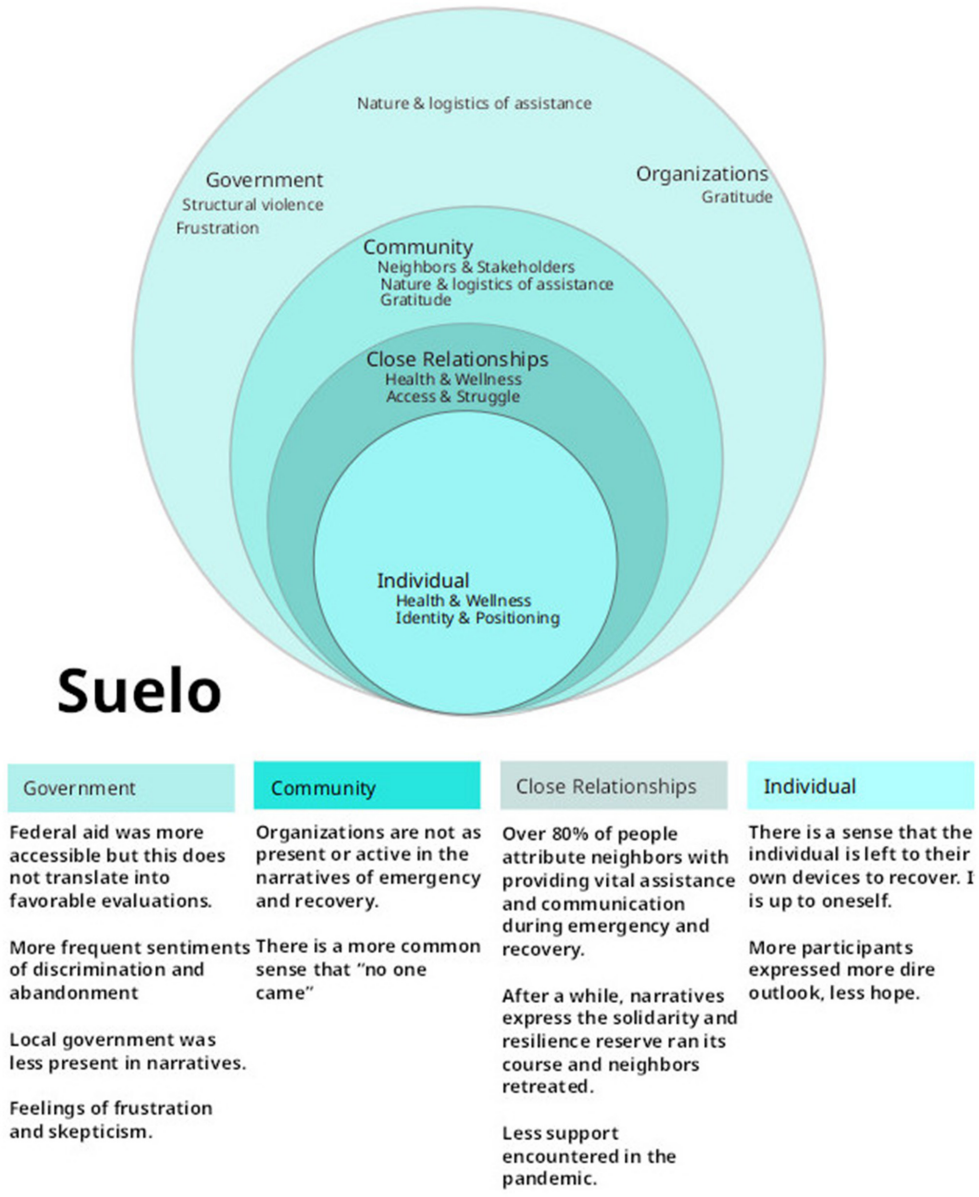
Ecosocial model of Suelo. Visual representation of actors, activities, and emotions associated with disaster recovery across four dimensions of interaction (Chopel et al., 2021).

For Aims 1, 2, and 3, we estimated panel and cross-sectional regression models and correlations. For Aim 4, we used a hybrid inductive/deductive thematic analysis technique outlined by Fereday and Muir-Cochrane (2006) to iteratively develop and test theory. All data collectors identified recurring or prevalent themes among all interviews they conducted, in the form of short memos. We transcribed 39% of interviews and conducted language analysis. Findings from the computer-assisted language analysis were triangulated with ethnographic observations and direct text analysis (Wignall and Barry, 2018) exploring tensions and contradictions, needs and agency. A careful review of text-based content surrounding top codes from the predetermined list and participant voice frequently used lists generated a third list exploring conceptual relationships between the two. Identified themes were defined and placed along the eco-social dimensions (see Figures 1, 2). Members of the field research team and transcription team joined in reviewing the interviews and analyzing salient themes. Next, we compared our qualitative findings between municipalities, and to our findings from Aims 1–3 to look for patterns, fit, and contradictions.

### 2.6. Engaging and disseminating the data

Local government representatives, research collaborators and participants were all invited to a 2-h data review meeting with the three co-principal investigators. Invitations were municipality specific. It was an opportunity to dispute or validate the data, eco-social models, and thematic analysis. At the meeting, the researchers shared graphic representations of results from both municipalities, revealing only which data belonged to their municipality, and referring to the other by the code name, either “Nube” or “Suelo,” accordingly. The meetings also allowed researchers to discuss potential recommendations to government agencies and gave room for residents to discuss their own takeaways for improving local preparedness. To incentivize participation throughout the value-chain of knowledge generation, participation in all meetings carried participation stipends for participants and research assistants.

Once the findings and policy recommendations were validated by participants, these were presented in a policy seminar, which included participants from federal government agencies (Federal Emergency Management Agency, FEMA) and local government officials (Chair of Health Committee in Puerto Rican Legislature), in addition to academic representatives (Chair of Department of Economics at the Interamerican University). The seminar was held at a local university and open to the public both in person and virtually, with students particularly encouraged to attend. A handout summarizing the findings and three data-informed action items was produced and disseminated at each event and online, in Spanish. Additional dissemination efforts in both languages were planned news media and professional meetings, including the following: the Natural Hazards Center Researcher Meeting, the Society for Applied Anthropology, a seminar hosted by the Puerto Rico League of Cities and followup meetings with government officials.

### 2.7. Challenges

Our mixed methods design relied on both secondary and primary data. Because analyses of the former were initially designed to inform where and how to collect the latter, careful time-management was needed. The research team had anticipated some challenges in gathering data due to the post-disaster context while in the middle of a pandemic and because of inconsistencies in data management across the US with regards to its territories. The coincidence of our research with the change in state government administration, generated additional delays that threatened the initial linear progression of research tasks.

As proposed, our research sought to replicate the analysis performed stateside by using the exact federal data sources for Puerto Rico. Though many federal sources, like the U.S. Census Bureau, US Department of Labor, US Economic Development Agency and data from the Center for Disease Control and SHELDUSTM are readily available online for the 50 states, they are not similarly available for US territories and treated differently across agencies. At times, municipalities were treated as counties, in other reports counties were regions of municipalities. Some reports were not available for territories at all, creating “data deserts,” which our experience suggested may be applicable to all US territories that are often left out of databases that tally states, but not territories. For the data we did get, we found it important to “trust but verify” all data. For example, we obtained the urban-rural measure from the National Center for Health Statistics that other researchers rely on to describe the degree of urbanity/rurality, in the knowledge that it greatly impacts many social, economic and health outcomes. Upon inspection, we realized that the categories, as applied, did not reflect a realistic understanding of Puerto Rico's geography; therefore, we created a population density measure that was imperfect but, we felt, better captured the true impact on infectious disease risk. To measure economic inequality we used the Gini coefficient for each municipality.

Going into the research, we knew many local sources of data were not readily available online and anticipated this might be problematic. This was especially relevant in accessing up to date local health data. COVID-19 incidence reports, for example, were provided daily online but datasets were not readily available for download nor organized into monthly totals by municipality introducing steps prior to data analysis. Personal appeals for specific or better data to government agencies were difficult due to the impact of post-election administration changes at the federal, territory, and municipal levels. With no easy online choices available, we decided to enter health and COVID-19 data manually in order to process it as needed.

Once in the field, the team had to balance the rapid nature of the research with a mindful approach to participants with a heightened risk for re-traumatization, anxiety, illness due to COVID-19, or fatigue. Inviting participants at random in public spaces had only moderate success. Still-fragile, intermittent telecommunication services added difficulty to coordinating interviews from afar. In the process of enlisting the collaboration of organizations, business leaders made it apparent that their operations were struggling to do more with fewer staff because in many cases they had just reopened and were trying to offset the pandemic-enforced closures. Other organizations were on limited schedules or had closed permanently due to COVID-19. “Free-time” to advance research was a luxury few could afford. In the face of these challenges, the team pivoted to work through social networks of trust.

Field research assistants from each study site took interviews as an opportunity for them to check-in and share stories with people they knew or were referred. This was an opportunity for the study to incorporate voices that would not be easily accessed in public spaces. Out of concern for accidental bias from occasionally familiar or emotionally engaged interactions, at least two members of the research team reviewed the interview transcripts to review interactions and to provide timely feedback. The observed trend was that when interactions were familiar, the process was more conversational but ultimately followed the questionnaire. Another strategy used to address bias, was the early sharing of data and findings with participants in town hall meetings.

### 2.8. Adaptations

In response to the delayed access to secondary data, the Co-PIs adjusted their research question. They also changed the rationale for selecting the municipalities for the case study (Aim 4) by looking a the relationship between poverty and distribution of funds (Aim 2) rather than informed by taking into consideration health data as well (Aim 3). This allowed the project to collect and analyze data as they became available, incorporating some immediately into analyses, and cleaning and storing others for future research opportunities.

To overcome ongoing trauma challenges and prevent fatigue, a large research team was recruited that included members of communities where we collected data. By recruiting participants and co-researchers at the same time and place, we both collapsed time between these activities and increased our team's contextual competency. To adapt to challenges presented by the pandemic, we created hybrid data collection procedures where some data were collected online, and some in person, while maintaining COVID-19 protections.

Between April and July 2021, FEMA held a Public Comment Period on Climate Change and Underserved Populations. In order to take advantage of this opportunity to share recommendations within the agency's timeframe, the Co-PIs preemptively developed and shared recommendations prior to the planned discussion and validation process with local stakeholders. The change in the order and process of collaborative review of findings and generation of recommendations reflected the Co-PIs priority on using findings to guide decision making in government disaster response policies. Once these findings initial findings were shared with FEMA, they were also presented, discussed, and expanded through the town hall meetings. This adaptation to the initial plan of events, reaffirmed the research teams' understanding that systemic change requires an iterative approach using a variety of engagement strategies.

## 3. Results

The study results are described in-depth in a report submitted to and published by the Natural Hazards Center of the University of Colorado, Boulder, available at: relationships-between-distribution-of-disaster-aid-poverty-and-health-in-puerto-rico. In this section, we provide a broad overview of our findings and reflect on the role of our methods in attaining the original study aims. Readers are invited to visit the report for more details, including tables and graphics in accompanying appendices.

### 3.1. Expected outcomes

The co-researchers anticipated their original research might find a positive relationship between disaster aid disbursed, accelerated growth in poverty and elevated economic inequality, at the municipal level. Quantitative analyses did reveal the expected patterns across all municipalities. This outcome was expected based on the research described in the introduction by researchers in the US. Our findings did support the expected outcome. In addition, we learned from primary qualitative data about potential reasons for the identified relationship between aid and economic outcomes. We further expected to find a pattern of relationships between COVID-19 positive cases and increased poverty and economic inequality. This expectation was based on decades of scholarship connecting economic inequality and poverty to poor health outcomes, across multiple causes of morbidity and mortality. Qualitative analyses contributed to identification of both potential pathways of causation and public health and policy recommendations.

When we look at the methodology used to complete the research, we had two expected outcomes. First, the Co-PIs expected the choice of using local research assistants would enable rapid data collection and ensure the experiences of marginalized populations were included. Second, research team hoped that through the rapid dissemination efforts we would see a growth in ownership of the knowledge generated in the form of interest and efforts that would result in shared authorship or the continued participation of participants from town hall meetings in the policy seminar. As for mid to long-term outcomes, we expected to see changes in how FEMA distributes aid and measure the success of their distribution efforts.

### 3.2. Findings

After accounting for the impact of changes in population, municipal poverty rates began increasing faster post the 2017 hurricanes. We found this increased rate was positively correlated with hurricane fatalities but not hurricane property damages. Moreover, poverty accelerated at a faster pace in areas that received more disaster aid. Case studies provided a disaggregated view of disaster aid showing its unequal distribution. Aid flowed, just not everywhere with ease, and more importantly, it was rendered out of reach for already marginalized populations. Its unequally distributed flow was in turn associated with increased health inequities. Interviews highlighted the post-disaster growth of extreme poverty and themes of structural violence. We found similarities between the two municipalities, such as an overall sense of violence from bureaucracy and governmental neglect, that were commonly connected to the economic and health costs of delayed and inequitable disbursement of government aid. We also found differences, such as a fluctuating resilience reserve where poverty was less extreme and more enduring hyperlocal support networks where extreme poverty created “everyday disasters” that required unending survival responses. Lastly, we found cumulative COVID-19 cases to be positively correlated with each of the following, ordered from strongest correlation to weakest: disbursed disaster aid, hurricane fatalities, economic inequality, and hurricane property damages.

In examining results of our methodological choices, we find mixed results. Recruiting research assistants from the municipalities studied gave us access to 75% of the primary data analyzed. By virtue of living, working or having relatives in the study sites, local research assistants were able incorporate participants experiencing economic duress, who felt they were sidestepped by a variety of disaster assistance efforts, and who were struggling to rebuild their lives. Some of these participants had multiple part-time jobs and had limited free time, others were not employed but did not have access to technology, had fear of contracting COVID-19 because of underlying conditions, or held a high distrust of strangers. The success of this early collaboration with research assistants did not guarantee, however, consistent, extended participation throughout the final stages of the study. Rapid dissemination activities did meet expectations, successfully engaging a variety of audiences including municipal government employees, residents, representatives from state and federal agencies, leaders of nonprofit organizations, professors, and students from a variety of fields. Though town hall meeting participants did not become repeat participants in the policy seminar, members of the research team were able to establish repeated meetings with FEMA employees to discuss findings and potential course of action to enhance equity in disaster recovery.

### 3.3. Advantages

The two main advantages of the study design were: (1) the interdisciplinary convergence framework and (2) the use of rapid data collection with rapid dissemination in order to contribute to real-time decision-making. Our team found that it was especially essential in disaster research to go beyond interdisciplinarity and actually build each other's capacities. Thanks to the interaction and know-how from each discipline represented, we were able to move quickly enough to capture perishable primary data while also utilizing available secondary data to guide research decisions. Primary data collection in a disaster recovery context is in itself challenging. The added anxiety of a global pandemic made interacting with strangers appear threatening. Intermittent electricity and internet access could offset this for some, but the digital divide marginalizes many experiences from being included. These filters to participation were offset by recruiting local research assistants to complement the data using their social networks to represent often overlooked populations. Our focus on disseminating and validating findings in the communities studied advanced not only the rigor of the study but also created a space for local actors to share ideas of how to more effectively coordinate assistance in their communities.

### 3.4. Limitations

While the rapid pace of the research was a strength for its applicability, it was a limitation when it came to the depth and breadth of our findings. The team had to cut some of the original research objectives when encountering obstacles in accessing secondary data in a timely manner and minor delays in receiving approval for ethical human subjects research protocols. While we were able to conduct two in-depth case studies in two sites selected with guidance from our quantitative findings, more time would have created the ability to contextualize the primary data within an in-depth review of secondary data from each municipality, providing even more information on potential causal pathways and potentially effective recommendations for public health and disaster aid distribution strategies.

Time constraints limited the ability of the team to engage with local communities and the findings in more meaningful ways. Participants, research assistants and municipal collaborators all faced a variety of competing interests and limitations. As it would happen, though we celebrated town hall meetings, some participants could not travel at night or coordinate assistance in time. Research assistants all faced a variety of opportunities and changes in priorities. Some students moved away, others found jobs, or internships. In the end, for a variety of reasons the invitation to continue their collaboration and become co-authors did not get the traction we had hoped. With more time, a variety of activities or means of engagement could have been divided to extend participation.

The overarching objective for the research was to conduct rapid research in order to inform policy and practice during the recovery period. Though the research responded to a special call for proposals by the Natural Hazards Center and received funding from two federal agencies, National Science Foundation and Centers for Disease Control and Prevention, many recommendations were for yet another agency: FEMA. As a result, though our work was federally funded, it was not “located” within the agency whose policies our recommendations addressed. This external positionality limits the ability to inform or account for change. The public invitation to share recommendations with FEMA via an online submission democratizes participation to reflect upon practice and incorporate data from research, but at the same time it invisibilizes the authors and interactions that inform changes.

Our rapid dissemination efforts attempted to facilitate systemic change by implementing a variety of engagement strategies: online webform submission, letters, individual meetings with municipal and agency leaders, town hall meetings, and a policy seminar that was both in person and virtually transmitted, but it is difficult to know the extent of the impact. The policy seminar organized by the research team provided a more visible chain of events that revealed institutional limitations to engage with local researchers. In subsequent interactions, as next steps and proposals for action were mentioned, local FEMA representatives informed the research team of their limited control over the decision-making process that made it possible to consult or provide solutions informing policies or practice. Contracting decisions are determined in central offices off the Island. Potential engagements would further require that contractors be able to provide professional services in a regional scale. The search for system-wide services and the generalizability of solutions for use in a multi-state region or nationwide, brought into focus potential systemic barriers to collaboration with local scientists and organizations. At the time of writing this chapter, beyond any rapid timeline, members of the research team are still in conversation with different government stakeholders exploring ways to increase awareness of how current aid distribution strategies contribute to greater social inequities and challenge disaster preparedness and public health.

## 4. Discussion

Our study aimed to replicate a quantitative approach to research as a first step to exploring relationships and patterns that could inform location-specific recommendations. As described in the methods section, we engaged in transdisciplinary analyses of our data, recruited research assistants from the case study sites to reach voices that may be repeatedly marginalized from aid and from representation in the public construction of knowledge, and adopted an iterative approach to communicating disaster research findings in order to advance its broader use. While our methods are replicable, we hope that future research replicating our methods may engender results that can change the way aid is distributed so that it contributes to increased economic equality and health equity. This would move the disaster response and recovery field toward identifying policy and programmatic interventions that practitioners could then strive to replicate.

Our team considers that research in a post-disaster context should ethically strive to be immediately useful to the current and local context where the research is conducted. Transdisciplinary teams with a willingness to break from disciplinary tradition, mixed methods, local engagement and rapid dissemination are key to this direction. In this section, we provide lessons and recommendations that emerge as a product of our reflection.

### 4.1. Lessons

#### 4.1.1. Rapid research needs to be agile

Architect Luis H. Sullivan coined the phrase “form follows function” in 1896. In the context of cascading and compounding disasters, it is essential to introduce both rapidity and agility to the methods design to ensure data collection and analysis can provide useful findings in timely fashion. We knew that with each passing day, the experiences, perspectives and ideas of potential research participants were likely to change. Memories of the disasters were prone to recede even more quickly than usual as earthquakes hit, followed by the COVID-19 pandemic. Fear of the unknown and safety occupied evermore brain activity, increasing the perishability of the data regarding Hurricane Maria disaster assistance. The research timeline and methods needed to fit the changing context in order to meet our research goals. Working within a convergence framework to investigate complex problems had the unexpected added benefit of providing our team with a variety of disciplinary approaches which proved helpful in adapting to the changing conditions in the field.

The form of our research team was developed according to function: the guiding research question required a public health expert, an economist, and an applied anthropologist. The public health researcher-practitioner helped to draw the connections between meteorological and fiscal disaster outcomes and health risks and outcomes. The economist contributed an understanding of the measures of poverty and economic equality, and statistical skills that our quantitative study aims relied upon. The applied anthropologist contributed not only experience and knowledge with qualitative ethnographic methods, but also connections to several communities, from local community case study sites to territory-level policy-makers. We knew this, and celebrated these complementary strengths, from the moment we came together.

Having these different skill sets enabled us to pivot quickly when we encountered a delay so that we could continue forward momentum and collect enough data to contribute to answering our research questions within less than half a year. Originally, we had planned to finish the first three aims and have the findings guide us in selecting the municipalities. Faced with delays, we adopted the public health orientation used in epidemiological studies around selecting units for comparison based on matching other potential impacting factors in an attempt to “isolate” the variables of interest. The investigation was informed from the field of anthropology through prioritizing relationships. The anthropologist visited several communities selected, relying on preliminary quantitative findings and matching communities on other characteristics.

In addition to the resourcefulness of a transdisciplinary team, it is important to be willing to pivot in response to changes and to collect data where and when you can. Our original research plan incorporated two major research questions: one focused on the independent variable of federal disaster response aid in dollars and the other focused on another form of disaster response and recovery resources, non-profit organizations, and specifically exploring the impact of their growth post-disaster employing a categorized count of organizations as the independent variable. Upon determining that matching the data sources of the study we were replicating Puerto Rico was, for some sources, impossible or would delay, and threaten, our ability to generate results in time, we decided to refocus our research design on disaster response aid with data that was already available. That quick and flexible pivot allowed us to complete the project within the specified timeframe, revealing useful findings and making disaster aid distribution recommendations for improved economic equality and health equity.

#### 4.1.2. Flexibility enhances participation

Participation was enhanced because we had local research assistants and a variety of ways, in-person, via telephone, or online, to collaborate with data collection. Research team members and study participants were all balancing their own path to recovery, navigating the impacts of compound crises. So long as project tasks could fit into their individual balancing act, they could ensure they contributed to advancing the project goals. As soon as the calendar of work grew in intensity, was limited by time and space, it lost flexibility and participation suffered.

#### 4.1.3. Build rigor and grow impact

We found that collecting and analyzing data iteratively and engaging local resources can enable rapid research that is rigorous and yields rich data. Our timeline did not allow us to collect all our primary data, analyze it all together, and then interpret it in a linear fashion. Neither were we able to convene and train an analysis team to follow a step-by-step process of transcribing, reading, defining codes, re-reading, re-defining codes, coding, grouping into themes, applying themes back to the text, identifying patterns, trends and connections between themes, and interpreting themes to apply meaning within the specific context of our research, in a linear way. Instead, we recruited community members to participate in data collection and analysis in an iterative way whereby data were collected, analyzed, and interpreted by people who were steeped in the context within which our research was situated. In this manner, we were able to leverage both the overlap of people's lived experience with the focused data and the overlap of each step with the other to extract deep and rich meaning from the primary qualitative data gathered, analyzed and interpreted in a very short timeframe. Finally, we also combined two separate objectives into one final set of activities: (1) to return findings to the communities where we collected data and (2) to engage local stakeholders in checking data, interpreting findings and discussing potential recommendations. This enabled us to stay aligned with our values as a research team around ensuring that people who contribute to data have access to the knowledge that is created using it, while also honoring the importance of member checking in qualitative research and leveraging the lived experience of community members and leaders in the translation of findings to potential recommendations for positive change.

Sharing data and preliminary analysis with stakeholders enabled more people to make better informed decisions along the way, within their timeframes. Initial letters and meetings with municipal employees about the concern motivating the research led to more active participation in the town hall meeting at one of the research sites. Individuals at the town hall meetings referenced past neighborhood structures and arrived at a consensus around the need to revitalize community level organizations to improve post-disaster recovery and risk mitigation efforts.

Though it is difficult to know what benefit was gained by FEMA from our response to their online call for recommendations, it undoubtedly contributed to the chorus of voices clamoring for improved equity considerations in the US federal disaster response and recovery activities. FEMA demonstrated a deepened focus on equity in their efforts as Goal #1 (of three) of the 2022–2026 Strategic Plan (published in December 2021) is: “Instill Equity as a Foundation of Emergency Management.”

### 4.2. Recommendations

As much of the literature we reviewed focused on rapid qualitative methods, we begin with some recommendations for rapid quantitative research. First, we recognize that even when using secondary data, data collection within a rapid research timeline can be challenging and such challenges must be planned for, with contingencies, to maintain the shortened timeframe. Once data are obtained, it can be helpful to envision how the data should be structured for most efficient analyses: cross section (multiple variables at a point in time), time series (a single variable at multiple points in time), or panel data/longitudinal (multiple variables at multiple time periods). Each structure brings its own set of challenges. As we brought multiple identification strategies into our models from different sources, the key challenge was determining whether the variables we had access to addressed the research questions clearly and if we used the proper econometric approach.

Designing a data dictionary may feel unnecessarily time-consuming, yet for our team it was essential and saved us time in intra-team communications. Your data dictionary table should include: all the links where the data were found, the date they were originally accessed, a description of each variable, why you chose that variable (whether or not there was a need to use proxies) and selected literature on the use of that variable, and their expected sign. There are three data structures: cross-section, panel data/longitudinal, and time-series. Data structure is key for your regression models to be estimated properly. If data is not arranged properly, your software may not be able to estimate the model (Wooldridge, 2019). There is a set of tools for every data structure and robustness tests allow researchers to determine the right fit (Greene, 2018). Finally, we recommend avoiding any variables with high standard deviation or variables that may be driving up the “biasedness” of the regression to avoid potential lack of clarity down the road.

In reviewing the literature, we found several similarities between the contexts that our qualitative data helped illuminate, trends that our quantitative data outlined, and relationships between causes and events, in scholarship exploring similar or related changes in Global South communities. While Puerto Rico is politically subsumed within the richest nation-state in the world, its economic context reflects characteristics of the poorest countries in the world. Therefore, when investigating changes in the economy and economic outcomes, it might make sense to look to methodologies developed within and for a Global South context. One such methodology is called the Livelihood Risks and Opportunities (LRO) framework for rapid research, and borrows from and combines elements of the impoverishment risks and returns and the sustainable livelihoods approaches to quickly measure changes in livelihoods across five elements (including financial, physical, and natural resources as well as social networks and skills). It was developed by Kabra (2016) for use in the wake of development-induced displacement, but can be applied to studying outcomes of major disruptions. While displacement is a major consequence of the disasters we studied, as demonstrated in a rigorous study of gentrification, displacement and economic segregation post-María in San Juan (Santiago-Bartolomei et al., 2022), it is not the only consequence and the methodology could help to compare livelihood outcomes between those who were displaced and those who were not but experienced a different set of risks and opportunities as a result of staying. Kabra suggests the study of such disruptions that are “development-induced, conservation-induced, and conflict-induced;” to those we suggest adding climate-induced.

Like our study, the framework uses mixed methods and engages with those who are impacted in a participatory way, though neither reach the standard of Community-Based Participatory Research where participants are engaged in research design decisions (Udoh et al., 2013; Chopel et al., 2021). Key to its applicability to studies of institutional responses to disruptions and their economic consequences (such as our own study), LRO also intentionally includes an analysis of policies and programs' promises and actual distributions. Importantly, social connectedness and social capital are incorporated into measures of risks, opportunities, and changes in each resulting from the disruptive event. The author points to the adaptability and flexibility of the method as well. Although its development borrowed from Participatory Rural Appraisal methods, the author states that the method can and has been used in diverse scales and across diverse geographies (urban, suburban, rural). By utilizing the framework to identify areas of measurement and quickly adapt a set of relevant, quantifiable measures, future rapid research in a post-disaster context could begin to address some of the many new research questions that our findings point to.

For example, other research teams have found across multiple contexts that, “Households with poor social networks suffered livelihood setbacks which many of them have not been able to recover from, leading to emergence of sharp social and economic differentiation in the post-relocation period” (Chopel et al., 2021). The parallels can be drawn to our study (Chopel et al., 2021) and the studies that inspired ours by Smiley et al. (2018) and Howell and Elliott (2019), and can potentially inform policy and programmatic directions for improving equity in aid distribution strategies and also inform future research directions. Furthermore, our qualitative findings affirm that the areas of focus that are prioritized by the LRO methodology can help to identify and describe the various factors at play, therefore creating meaningful findings that can inform interventions to reverse the identified trends. For example, the methodologies “highlight the role of state institutions and processes as well as the affected people's own coping strategies for livelihood reconstruction” (Kabra, 2016). Our findings around the differential community coping strategies between two towns that were both experiencing community-level poverty in the disaster recovery context, but with a slightly different starting point in terms of pre-existing poverty and economic (in)equality, demonstrate the importance of not only considering policies and institutions when studying their impacts, but also of understanding and taking into account the people they are affecting, and the different ways in which their unique contexts can shape similar policies into very different outcomes.

This body of scholarship, and adaptation of similar methodologies, can also help to extend our understanding of the longer-term impacts on poverty and economic inequality that our research pointed to in the more immediate recovery period. For example, Kabra (2016) found that the change event led to negative outcomes in perceived creditworthiness and prolonged reduced access to credit. Given that credit, like wealth and income, is already very unevenly distributed, it is likely that the increased poverty and economic inequality that was connected to the post-Hurricane Maria aid distribution strategy in Puerto Rico will also impact the longer term credit options of people who live in marginalized communities across Puerto Rico. To interrupt further concentration of poverty, it would be worthwhile to study this aspect of disaster recovery and rapidly disseminate results for immediate translation into policy and programmatic interventions. It is essential that future research be conducted as rapidly as possible, to ensure perishable data are gathered but also because the rapid pace of changes, and the growing risks to livelihood and health that come with them, make these questions urgent, as a matter of life and death.

Reflecting upon rapid dissemination activities, we find that the early sharing of data and findings, did engender expressions of ownership of the data and public expressions of how to apply it. Municipal staff and residents to recognized the importance of strengthening social networks to improve readiness, response, and recovery at the local level. The lively exchange during and after the policy seminar is another example of collaborators engaging with knowledge being discussed.

Local partnerships at recovery sites are best to lead rapid response, research, and dissemination, and have the potential to enable rapid learning and quality improvement in disaster recovery. As discussed in the limitations, members of the research team identified bureaucratic barriers with the potential to systemically exclude local partnerships. To minimize this potential, we recommend for federal agencies identify segments of the operational budget for local contracting. This is already done for post-disaster debris removal, construction or field personnel, but could be done for training and evaluation services as well. Strategically designed pilot programs or calls for proposals can be developed to foster local collaborations in the generation of knowledge allowing agencies to rapidly respond to different disaster contexts and key regional differences. This practice has already demonstrated success when used by the Natural Hazard Center, the National Science Foundation, the Center for Disease Control and the Environmental Protection Agency. Such a proactive engagement with local scientists and organizations has the added benefit of contributing to decolonizing recovery efforts and disaster-informed science. A similar recommendation is raised within the original research that suggest inequitable impacts of current aid distribution patterns could be reduced if federal agencies were able to pilot new distribution or engagement strategies to respond to the rapidly changing post-disaster context.

Inclusive practices need to be designed to address the value-chain of knowledge generation, from research design to public dissemination. Alternatives for meaningful authorship and credit should be defined and be subject to review. Changing protocols within a rapid timeline is perhaps easier early on, but gets increasingly more challenging as due dates appear on the horizon. In the initial report, after noting that the original path to authorship had not rendered anticipated results, contributorship was used as the default mechanism for inclusion of all collaborators. Intent on corroborating initial assessment of why and how participation tapered off in the final stages of the rapid research, the Co-PIs invited the field research team to review and discuss the reflections in this chapter. Outside of rapid schedule deadlines, the present reflection benefited from tasks and responsibilities defined with added mindfulness to competing schedules. Flexibility aided inclusive authorship.

In our changing world, where disasters last longer and are more frequent, making almost all natural hazards that hit unprepared human settlements result in compounding or cascading disasters, rapid research is becoming more and more important. As Kyrkjebo et al. (2021) argue in their description of Rapid Research Assessment used in New York City for COVID-19 response planning, “organizational sense-making is a usable climate service.” Future researchers should seek to incorporate or inform policy makers as early and often as possible, to ensure that the questions and the findings are usable and timed right (Kyrkjebo et al., 2021). Just as researchers are likely to adapt our methods, approach, and dissemination strategies to the increasing and transforming needs, government agencies should pivot their strategies to quickly integrate lessons learned from research. For example, it is clear from our experience that greater inter-agency collaboration is needed to ensure funded research has a feedback path that feeds into the decision-making of multiple interconnected policy-making and policy-implementing agencies.

Given that our research illuminated some unintended negative consequences of public disaster response aid, the disaster research community should also apply rapid research methodologies toward the support of the business community as crucial actors both before and after natural disasters. Saleem et al. (2008) have developed a model for pre- and post-disaster business continuity that could be both useful and adaptable. However, we caution against adapting a tool without ensuring that the research team includes people who survived the disaster and are fluent in the local context. In our team, experience and expertise were key to determining when it was appropriate to give up on seeking data and pivot to focus on data we ourselves could collect and analyze rapidly and iteratively.

## 5. Conclusions

Rapid research creates opportunities to make data-informed program and policy adjustments when they can be most impactful. In our use of rapid research methodologies, our goal was to generate knowledge about relationships that impacted disaster recovery in order to facilitate change in institutional aid disbursement policies. We used combined recruiting of local collaborators in data collection and analysis, with community-level dissemination. The early and iterative dissemination grew trustworthiness in our findings and enabled us to hone findings further before presenting to policy makers and media. Our mixed method findings demonstrated that, in the case of Puerto Rico, unless equity is conscientiously aimed for, aid is likely to follow existing, worn paths of power, privilege, and marginalization to amplify existing inequities rather than creating new paths for improved equity and a just recovery. Alex Steffen, a futurist particularly concerned with climate change and disasters, coined the term *predatory delay*. He defined it as “the blocking or slowing of needed change, in order to make money off unsustainable, unjust systems in the meantime.” The “in order to,” or the connection between the money being made from unsustainable or unjust systems may not be as clear as it is phrased here. What is clear, however, is that in a post-disaster context, delay kills people, and it kills poor and working class people more, and more quickly. This connection was demonstrated by several studies of the contended number of excess deaths that could be attributed to Hurricane Maria specifically within Puerto Rico (Cowan, 2022).

A quick glance at the amount and pace of aid sent to Texas and Florida, in comparison to the amount and pace of aid sent to Puerto Rico, and juxtaposed with the number of injuries and deaths experienced in these places during the same time period, makes clear that delay was, and continues to be, *predatory* in Puerto Rico. Willison et al. (2019) found that, “within the first 9 days after the hurricanes hit, both Harvey and Irma survivors [in Texas and Florida] had already each received nearly US$100 million in FEMA dollars awarded to individuals and families, whereas Maria survivors [in Puerto Rico and the Virgin Islands] had only received slightly over US$6 million in recovery aid.” Framed within a national context, the treatment of Puerto Rico by the federal government in its disaster aid disbursement reinforces demonstrated inequitable treatment and outcomes for Latinx/Latine communities across all other parts of the US. Whether that is intentional or not is unknown. Regardless of intent, however, we do know that an information gap contributed to the delay (Goldwyn et al., 2022). The challenges experienced by our team in accessing secondary data is an example of such.

Our team believes that rapid research has the potential to contribute to reducing predatory delay and bring attention to mechanisms that reproduce systemic racio-colonial inequities. Our quantitative findings identified a pattern that we see globally, both in the Global North (Smiley et al., 2018; Howell and Elliott, 2019) and in the Global South (Islam and Walkerden, 2015; De Alwis and Noy, 2019). Our qualitative findings underscore the variability in the relationships between those outcomes, not only between countries but even between municipalities within the Puerto Rico. Therefore, we underscore the importance of using rapid research methodologies to both look for larger patterns found elsewhere while also increasing understanding of the ways that the hyperlocal context changes and mediates pathways, via differing intermediary outcomes and other influencing factors. We conclude by reiterating our main recommendation: aid disbursement strategies must be purposefully designed to proportionally meet needs, measured *not only* in terms of severity of the disaster *but also* accounting for preexisting population vulnerabilities created by a system that marginalizes poor and working-class communities, and communities of color. We are convinced that rapid research can and will inform that strategy, making it more specific and more effective in its design and implementation.

Both the media and policy makers pay closer attention to research on current events. Hence, our recommendations are to fund and support more rapid research and to work early and iteratively to enable more stakeholders to engage in the process. The more rapid research and rapid dissemination we do, the better we will get at it, and the more accustomed community leaders, policy makers and program designers will become to using data to inform their decisions. Rapid research can be an important tool to correct economic inequality and improve the lives of people forced to live on the margins of our society.

## Data availability statement

The data analyzed in this study is subject to the following licenses/restrictions: available upon request to corresponding authors. Requests to access these datasets should be directed to lgorbea@prpassworkshop.org.

## Ethics statement

The studies involving human participants were reviewed and approved by Ethical and Independent Review Services. The patients/participants provided their written informed consent to participate in this study.

## Author contributions

LG, ACh, and AF were the co-principal investigators that proposed and were responsible for completing the research project. ACh designed the study, ensured that the methodology fit the theoretical framework, advised on data collection and analysis, and led the development of the initial draft of the manuscript. LG brought the teams together, led the primary qualitative data collection and analysis, and collaborated in the writing of initial draft and led the final revision process. AF led the quantitative modeling and gathering of secondary data. LB, GR, NP, ACa, JM, LL, and PS were members of the research team led by LG that assisted in gathering data, joined in the reflection of the methodology and provided a critical review of the manuscript.

## Footnotes

^1^The field research team was designed to include content experts in poverty, inequality and disasters, and experiential experts in the communities studied. We wish to acknowledge the assistance of our research associates and site consultants: Lorena Bonilla Marrero, Anohiska Cardona, Linda Colón, Junia Howell, Luis La Santa, Juneilis Mulero Oliveras, Nicole Pecci Zegrí, Gerardo Rivera Figueroa, Ana Hilda Rodríguez, Paola Sánchez, Daisy Vázquez, and Paulina Meléndez Olivera.
